# Bilateral Postprocedural Rhinitis After Intravenous Sedation With Supplemental Nasal Oxygen (PRAISE SNOG) After Cataract Surgery

**DOI:** 10.7759/cureus.12452

**Published:** 2021-01-03

**Authors:** Philip R Cohen, Daniel J Coden, Razelle Kurzrock

**Affiliations:** 1 Dermatology, San Diego Family Dermatology, National City, USA; 2 Ophthalmology, La Jolla Laser Vision & Eye Center - Acuity Eye Group, La Jolla, USA; 3 Center for Personalized Cancer Therapy, University of California San Diego Moores Cancer Center, La Jolla, USA

**Keywords:** cannula, cataract, extraction, nasal, oxygen, rhinorrhea, rhinitis, sneeze, sneezing, surgery

## Abstract

Rhinitis is classified as allergic or nonallergic. It presents with nasal congestion, nasal pruritus, posterior nasal drainage, rhinorrhea, and/or sneezing. During short procedures, nasal cannula administration of supplemental oxygen may be utilized to prevent hypoxia. Postprocedural rhinitis after intravenous sedation with supplemental nasal oxygen (PRAISE SNOG) - a noninflammatory variant of nonallergic rhinitis - has been observed in colonoscopy patients. Symptoms (sneezing and/or rhinorrhea with or without tearing) typically begin during emergence from sedation and persist for hours to days before resolving. A 66-year-old woman developed bilateral PRAISE SNOG following cataract extraction; her bilateral symptoms of nasal pruritus, rhinorrhea, and sneezing began immediately after awakening from sedation and spontaneously resolved within 24 hours. Mucosal irritation by the nasal cannula prongs that deliver the oxygen is a postulated pathogenesis for postprocedural rhinitis. Modification of the nasal prong composition (by using a soft silicon-based material), placement (by insertion prior to the induction of sedation and by not impinging on the nasal mucosa), and length (by trimming from 10 to two millimeters) are possible actions that might be initiated in order to prevent PRAISE SNOG.

## Introduction

Rhinitis - a disorder of nasal mucosa inflammation that clinically manifests as nasal congestion, nasal pruritus, posterior nasal drainage, rhinorrhea, and/or sneezing - is either allergic or nonallergic in etiology. Allergic rhinitis is caused by environmental allergens that elicit an immunoglobulin E-mediated response. Nonallergic rhinitis can be inflammatory (nasal polyp associated, nonallergic rhinitis with eosinophilia, or postinfectious) or noninflammatory (hormone-induced, idiopathic which is also referred to as vasomotor, medication-induced, or systemic disease associated) [1-5].

Supplemental oxygen is utilized during short procedures to prevent hypoxia. It is usually provided using a nasal cannula. Postprocedural rhinitis after sedation with supplemental oxygen administered via a nasal cannula during endoscopy - a noninflammatory variant of nonallergic rhinitis - has been observed [6, 7].

A 66-year-old woman had cataract surgery and received supplemental oxygen during the procedure via nasal cannula. Postoperatively, she experienced severe sneezing and rhinorrhea, which spontaneously resolved within the next 24 hours. The features of postprocedural rhinitis after intravenous sedation with supplemental nasal oxygen (PRAISE SNOG) are reviewed.

## Case presentation

A healthy 66-year-old woman had a right eye cataract. Testing for Coronavirus disease 2019 (COVID-19) was negative both two weeks and two days prior to surgery. She had no symptoms of upper respiratory infection and she had no history of photic sneezing.

Cataract extraction was performed. Intravenous sedation was achieved and maintained with propofol. Postauricular and peribulbar local anesthesia (consisting of an equal volume of two percent lidocaine and 0.5 percent plain bupivacaine with hyaluronidase at room temperature) was performed. Supplemental oxygen was administered at four liters per minute using a nasal cannula with 10 millimeter nasal prongs. The duration of the procedure was 20 minutes.

Immediately on awakening from anesthesia, she had bilateral severe sneezing and profuse rhinorrhea; there was also pruritus. Except for the acute onset of non-allergic rhinitis, she had neither fever nor headache. She used more than 300 tissues (three boxes of 100 tissues each) within the first postoperative hour.

**Figure 1:**
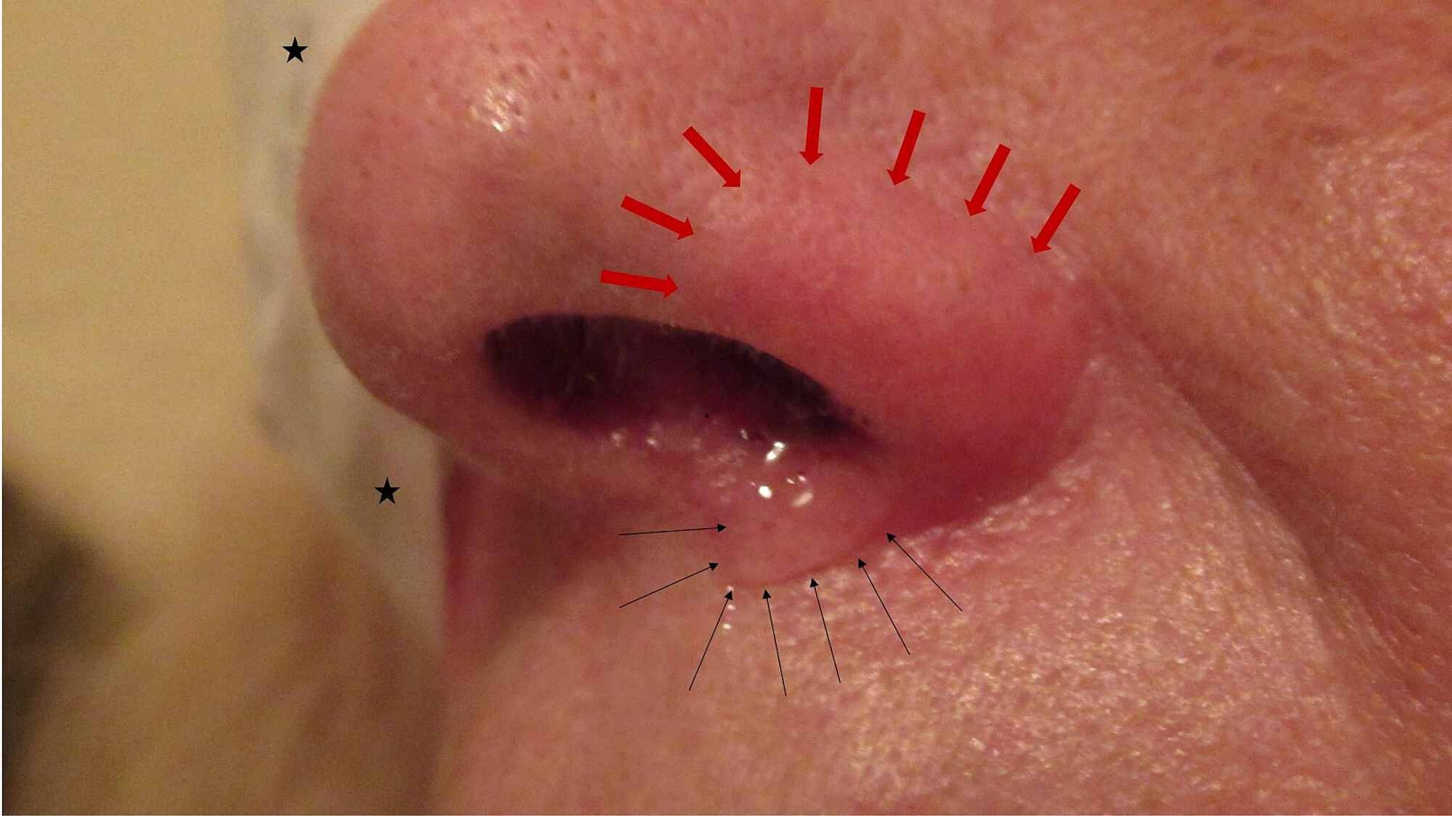
Postprocedural rhinitis after intravenous sedation with supplemental nasal oxygen (PRAISE SNOG) The left side of the nose of a 66-year-old woman shows erythema of the nasal ala (red arrows) and rhinorrhea from the nostril onto the upper lip (black arrows). Immediately after awakening from sedation following cataract surgery on her right eye (black asterisks on eye patch), she developed profuse bilateral rhinorrhea and sneezing. The PRAISE SNOG was less severe on the right side six hours later and had resolved on the left side within 24 hours.

Within six hours, the rhinitis symptoms on her right side had diminished. However, the sneezing and rhinorrhea from the left side of her nose and nostril persisted. In addition, there was an erythema of her left nasal ala (Figure 1). All of the left-sided symptoms resolved spontaneously within 24 hours postoperatively and have not recurred.

Approximately two years earlier, she had a left cataract extraction performed by the same ophthalmologist. The local anesthesia and intravenous sedation were the same. However, supplemental oxygen had not been used during the surgery and she did not experience PRAISE SNOG.

## Discussion

Sneezing can occur during drug-induced, such as propofol, sedation for endoscopy and oculoplastic procedures [8-11]. The photic sneeze reflex, an autosomal dominant inherited condition, can be elicited by numerous stimuli, including bright lights and periocular injections [9-11]. This reflex can be suppressed by using either fentanyl, antihistamine, or dexmedetomidine prior to the propofol injection [11].

Nonallergic rhinitis (sneezing and/or rhinorrhea with or without tearing) following endoscopy (which included both esophagogastrodenoscopy and full-length colonoscopy) was originally reported in a study involving 836 patients; all of the patients had received intravenous sedation and supplemental oxygen [6]. Subsequently, unilateral rhinorrhea and sneezing after upper gastrointestinal endoscopy under intravenous propofol sedation with supplemental oxygen administered via a nasal cannula was described in a case report [7]. In addition, this phenomenon has been mentioned on the internet by individuals who develop rhinitis immediately after awakening from sedation following a colonoscopy for which they received intravenous sedation and supplemental nasal oxygen; the comments - currently from 183 patients as of December 22, 2020 - can be found as Google blog posts accompanying an article on “Sneezing, runny nose and tearing after colonoscopy” [12].

We suspect that the incidence of postprocedural rhinitis after intravenous sedation with supplemental nasal oxygen may be greater than implied by the number of publications on the condition. To simplify the designation of this adverse event, an acronym has been introduced which emphasizes the salient features of this procedural complication: PRAISE SNOG. Praise is to express warm approval, and snog is a passionate kiss. “PRAIS” represents the first letters of the following words: postprocedural rhinitis after intravenous sedation. The “E” is the second letter of “sedation”. “SNO” represents the first letters of the following words: supplemental nasal oxygen. And “G” is the fourth letter of “oxygen”.

Li et al. evaluated the development of PRAISE SNOG in three groups of endoscopy patients who received intravenous sedation and supplemental oxygen either via nasal cannula with 10 millimeter prongs (294 individuals), a trimmed nasal cannula with two millimeter prongs (268 individuals), or nasal mask (274 individuals); the mean duration of the procedure was less than 19 minutes. Rhinitis symptoms developed in 7.1% of the nasal cannula group (presenting as either profuse rhinorrhea - with sneezing [10 patients] or alone [eight patients] or with sneezing and tearing [one patient] - or sneezing [two patients]) and 0.4% of the trimmed nasal cannula group (presenting as rhinorrhea in one patient); none of the nasal mask patients experienced PRAISE SNOG. However, the investigators observed that the incidence of transient hypoxia lasting less than 30 seconds during the procedure was lower in the nasal cannula group (3.1%) as compared to either the trimmed nasal cannula group (7.8%) or the nasal mask group (6.6%) [6].

Rah and Merkel reported a 65-year-old woman who developed left-sided severe sneezing, clear nasal discharge and tearing immediately upon awakening from the propofol sedation used during her 17-minute upper gastrointestinal endoscopy. She had received supplemental nasal oxygen at three liters per minute using a soft and flexible silicon-based nasal cannula, which has 10 millimeter prongs. She treated her symptoms with over-the-counter antihistamines and a nasal decongestant spray; they resolved within five days. Six months later, she subsequently had a colonoscopy using propofol sedation and supplemental oxygen with the same nasal cannula; however, the oxygen was administered via an oral route, and she did not develop PRAISE SNOG [7]. 

Albeit rare, severe adverse events have been associated with nasal cannula supplemental oxygen. Smoking while receiving home oxygen therapy via nasal cannula has not only resulted in facial burns from the associated fires but also death [13]. In addition, a nasal cannula supplemental oxygen malfunction resulted in a severe nasal frostbite injury in a woman of more than 90 years of age; the investigators postulated that a dysfunction of the oxygen pressure regulator, allowing a sudden decompression of the compressed oxygen, resulted in the low temperature of delivered oxygen and subsequent injury [14].

Mechanical irritation of the mucosa by the nasal cannula prongs is speculated as the etiology for the rhinitis symptoms during the endoscopy-related sedation; the contributory role of oxygen in the pathogenesis of PRAISE SNOG remains to be determined [6]. The prong tips of the nasal cannula were capable of eliciting sufficient nasal mucosal injury to result in nasal vestibule mechanoreceptor stimulation. Subsequently, this activated not only the nasonasal reflexes (involving cholinergic efferent and afferent responses) but also the local axonal reflexes (with symptomatic responses on both the ipsilateral and contralateral sides of the nose) [7]. 

Several interventions have been suggested that might prevent PRAISE SNOG from occurring (Table 1) [6, 7]. Li et al. recommended shortening the nasal cannula prongs or using a nasal mask [6]. Based on their proposed pathogenesis of PRAISE SNOG, Rah and Merkel suggested using an anticholinergic nasal spray in patients who develop this adverse sequela [7].

**Table 1:** Interventions to potentially prevent postprocedural rhinitis after intravenous sedation with supplemental nasal oxygen (PRAISE SNOG) ^a^If the nasal prongs are causing burning, discomfort, itching, and/or painful sensations, an awake patient shall be able either to request the clinician to readjust the prongs or move the prongs themselves to a more comfortable position. ^b^Vigilance is especially important after changing the patient’s position or moving the patient.

Intervention	Reference
A nasal cannula with soft silicon-based nasal prongs should be used.	[7]
Around the head and neck, the nasal cannula should be noncompressible.	[7]
Impingement of the nasal prongs against the mucous membrane of the nose should be avoided by the clinician.	[7]
Place the nasal cannula before the induction of sedation.^a^	[7]
Trim the prongs of the nasal cannula or use a nasal mask.	[6]
When using a nasal cannula, the clinician needs to be vigilant.^b^	[7]

Our patient developed PRAISE SNOG after an ophthalmologic procedure: cataract surgery. The intravenous sedation she received and the duration of her procedure were both similar to those occurring in patients undergoing endoscopy. Her rhinitis appeared immediately after emergence from sedation. Initially, her symptoms of rhinorrhea and sneezing were severe and bilateral; however, within six hours, the symptoms on the ipsilateral side as her surgery had improved, and within 24 hours, the rhinorrhea and sneezing had resolved.

## Conclusions

Postprocedural rhinitis following intravenous sedation and supplemental nasal oxygen has been observed in colonoscopy patients. A woman is described who developed bilateral PRAISE SNOG that began immediately after awakening from sedation following cataract extraction; her symptoms spontaneously resolved within 24 hours. A proposed etiology for rhinitis is attributed to the mucosal irritation by the nasal cannula prongs that deliver the oxygen. Interventions to potentially prevent PRAISE SNOG include modification of the composition (by using a soft silicon-based material), the placement (by insertion prior to sedation induction and by not impinging on the nasal mucosa), and the length (by trimming from 10 to two millimeters) of the nasal prongs used to deliver the oxygen.
